# Coexisting Prolactin-Secreting Macroadenoma, Hypopituitarism and Type 1 Diabetes Mellitus in a Young Adult Male Patient

**DOI:** 10.7759/cureus.20474

**Published:** 2021-12-16

**Authors:** Emmanuel Ssemmondo, Anis Abobaker, Jonathan Thow

**Affiliations:** 1 Endocrinology and Diabetes, York Teaching Hospital, National Health Service (NHS) Foundation Trust, York, GBR; 2 Rehabilitation and Care, Mid Cheshire Hospitals, National Health Service (NHS) Foundation Trust, Crewe, GBR

**Keywords:** insulin requirement, young adult male, hypopituitarism, prolactinoma, type i diabetes mellitus

## Abstract

The association between type 1 diabetes mellitus (DM) and pituitary prolactinoma is rarely reported in the literature. Herein we present a 23-year-old male patient with co-existing type 1 DM, pituitary macro-prolactinoma and panhypopituitarism secondary to mass effect. The patient presented with generalised symptoms including fatigability, cold hands, decreased insulin requirement secondary to frequent hypoglycaemic episodes two weeks following the diagnosis of type 1 DM. Initial blood workup showed evidence of possible secondary hypothyroidism. The full pituitary profile screen showed profound anterior hypopituitarism with a prolactin level of 67,168 miu/L (normal range 86-324 miu/L). Pituitary MRI showed macroadenoma, 37mm in size, with a suprasellar cystic extension but no chiasmal compression. It was treated medically with cabergoline and a repeat pituitary MRI after eight weeks of initiating treatment showed a 4-mm reduction in the size of the adenoma, and prolactin level decreased to 6,794 miu/L. The case was discussed at the Neurosurgical MDT and the outcome was to continue to monitor while on cabergoline. This case report is the second in the literature, which documented the association between type 1 DM and pituitary prolactinoma in adolescents. These patients might not present with the classical clinical features of hyperprolactinemia, and instead, they present with frequent hypoglycaemia and decreased insulin requirement due to secondary adrenal insufficiency as a consequence of the mass effect of the prolactinoma. Furthermore, non-specific symptoms, such as generalised tiredness and fatiguability, despite reasonable blood sugar control, in young patients with type 1 DM should trigger screening for all anterior pituitary hormones to exclude hypopituitarism.

## Introduction

Although the association between type 1 diabetes mellitus (DM) and hypopituitarism has been well-established and reported in the literature, the association between type 1 DM and prolactinoma is rarely reported. One case report described a 16-year-old female patient with a previous history of type 1 DM who presented with classical manifestations of hyperprolactinemia (primary amenorrhea, galactorrhoea and puberty developmental arrest) [1]. Pituitary MRI confirmed the diagnosis of pituitary microadenoma. Another report presented a 58-year-old male patient with a past medical history of insulin-dependent DM presented with impotence caused by pituitary macroprolactinoma [2]. In this case, we report a young adult patient who presented with co-existing type 1 DM, hypopituitarism and macroprolactinoma without the typical clinical features of hyperprolactinemia.

## Case presentation

**Figure 1:**
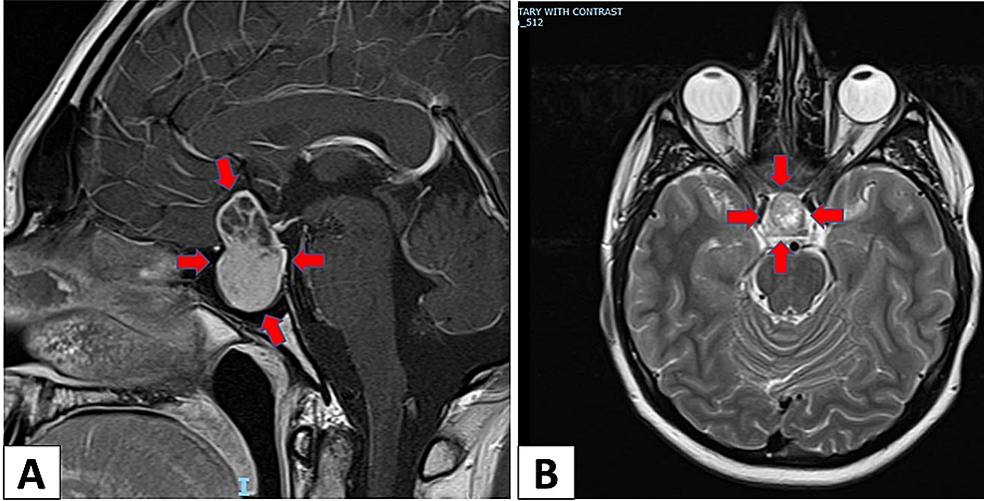
Sagittal (A) and axial (B) sections of brain MRI show 37mm pituitary macroadenoma with suprasellar cystic extension (red arrows)

Herein we present a 23-year-old British man without previous significant past medical history who was admitted to the hospital with Diabetic ketoacidosis (DKA). On admission, his PH was 7.28 with blood ketones of 6.7 and a blood glucose of over 30mmol/L. He was treated with intravenous insulin and fluids as per the local DKA management protocol, and subsequently discharged on a basal-bolus insulin regime. At discharge, he was taking 20 units of insulin glargine (abasaglar) once a day and insulin aspart (Novorapid) with meals (given as six units with breakfast, eight units with lunch and six units with the evening meal). Results for anti-GAD, IA-2, and zinc transporter antibodies were all positive, confirming the diagnosis of type 1 DM. Nearly two weeks later, he was reviewed in the outpatient clinic. Although he reported feeling much better, he still complained of feeling relatively tired and having cold hands. At the same clinic review, he reported frequent hypoglycaemic episodes. He had a review by the dietitian and carbohydrates counting was rediscussed. The ratio was changed from 1:10 (one unit of insulin for every 10 grams of carbohydrates) to 1:20 (one unit of insulin for every 20 grams of carbohydrates). His background insulin was reduced from 20 to 10 units. He was also started on flash glucose monitoring with free style libre 2. Thyroid function tests were done to exclude hypothyroidism. Results from this showed normal thyroid-stimulating hormone (TSH) level despite low T3 and 4 levels, which raised the suspicion of secondary hypothyroidism, and prompted screening of the full pituitary profile. Pituitary function tests showed profound anterior hypopituitarism, with a very high prolactin level (see results in Table 1). He was started on cabergoline 500 microgram weekly and hydrocortisone. He subsequently had an urgent visual field assessment and MRI head. Visual field examination was normal and the MRI head showed a pituitary macroadenoma, 37mm in size, with a suprasellar cystic extension but no chiasmal compression (Figure 1). He had two further reviews and the follow-up blood tests as shown in Table 1. He was discussed at the Neurosurgical MDT and the outcome was to continue to monitor while on cabergoline. MRI head done eight weeks following initiation of cabergoline showed a 4mm reduction in the size of the pituitary adenoma.

**Table 1:** Summary of the biochemical blood results TSH: thyroid-stimulating hormone; FSH: follicular stimulating hormone; TPO: thyroid peroxidase; IGA: immunoglobulin A; ACTH: adreno-corticotropic hormone; IGF-1: insulin-like growth factor 1; HBa1c: haemoglobin a1c

		Admission	Outpatient follow-up visits				
Biochemical parameter (units)	Lab normal range	27.07.2021 (Admission)	10.08.2021	13.08.21	09.09.2021	14.09.2021	05.10.2021
T4 (pmol/L)	11-22		7	7		10	12
TSH (mU/L)	0.27-4.2		6.2	6.1			2.0
T3 (pmol/L)	3.1-6.8					2.4	2.6
Cortisol (random) (nmol/L)	133-537		38	60	375		
Prolactin (miu/L)	86-324		67,168	63,420		9,173	6,794
Luteinising Hormone (iu/L)	1.7-8.6		1.3	1.1		1.4	
FSH (iu/L)	1.5-12.4		<1.0	<1.0		<1.0	
Testosterone (nmol/L)	8.64-29		0.85	0.81		1.59	1.39
TPO antibodies (ku/L)			<15				
IgA (g/L)	0.8-2.8		2.19				
Plasma ACTH (ng/L)	0-46			20			
IGF-1 (nmol/L)	17.7-45.1			14.6		18.4	20.3
HBa1c (mmol/mol)		102					61

## Discussion

The main reported aetiologies of hypopituitarism in patients with type 1 DM include autoimmune lymphocytic hypophysitis, radiational-induced, pituitary hypoplasia and spontaneous necrosis of the pituitary gland [3-8]. In our case, hypopituitarism was due to the mass effect of the macroprolactinoma. As with previously reported cases, after the diagnosis of type 1 DM and starting insulin treatment, our patient presented with recurrent hypoglycaemia and an unexplained decrease in insulin requirement after hospital discharge [3,5,7]. The hypoglycaemic episodes recorded following hospital discharge may be indicative of hypoadrenalism secondary to adrenocorticotropic hormone deficiency. Interestingly, in a 70-year-old female Japanese patient with a previous history of panhypopituitarism, the diagnosis of diabetes was uncovered after starting steroid treatment for secondary adrenal insufficiency [9]. In fact, there are a few learning points from our case. Firstly, the association between type 1 DM and prolactinoma is rare. These patients might not have the classical clinical features of hyperprolactinemia, and instead, present with non-specific features due to underlying hypopituitarism secondary to mass effect. Secondly, the presence of secondary adrenal insufficiency might mask the diagnosis of DM or lead to recurrent episodes of hypoglycaemia and unexplained decrease in insulin requirement in patients who were recently diagnosed with type 1 DM. This should prompt screening for adrenal insufficiency and panhypopituitarism. Lastly, non-specific symptoms, such as generalised tiredness and fatiguability, despite reasonable blood sugar readings, in young patients with type 1 DM should trigger screening for all anterior pituitary hormones to exclude hypopituitarism.

## Conclusions

To the best of our knowledge, this is the second case report which documents the association between type 1 DM and pituitary prolactinoma in patients under the age of 30-year. The main message is to consider the full screening of anterior pituitary hormones in type 1 DM patients who present with frequent hypoglycaemic episodes and decreased insulin requirements, or those who developed generalised non-specific symptoms despite reasonable blood sugar control.
